# Circumscribed Palmar Hypokeratosis: Case Report of an Underdiagnosed Disease

**DOI:** 10.5826/dpc.1101a109

**Published:** 2021-01-29

**Authors:** Judit Algarra-Sahuquillo, María-del-Mar Pestana-Eliche, Eva Fagundo-González, José-María Ramírez-Conchas, Rosa-Nieves Rodríguez-Rodríguez

**Affiliations:** 1 Dermatology Department, Hospital Universitario de Canarias, La Laguna, Tenerife, Spain; 2 Pathology Department, Hospital Universitario de Canarias, La Laguna, Tenerife, Spain

**Keywords:** hypokeratosis, stratum corneum, palms, keratinocyte

## Introduction

Circumscribed palmar hypokeratosis is a rare asymptomatic condition consisting of focal thinning of the stratum corneum. Little is known about this entity, and only a few cases have been described.

## Case Presentation

**Figure 1:**
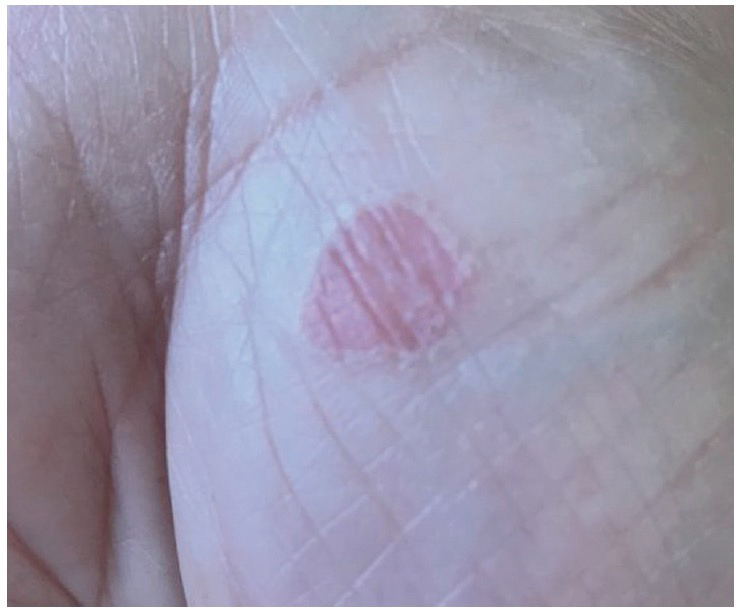
Clinical image of a single well-demarcated, erythematous plaque in the thenar eminence of a 63-year-old woman.

**Figure 2:**
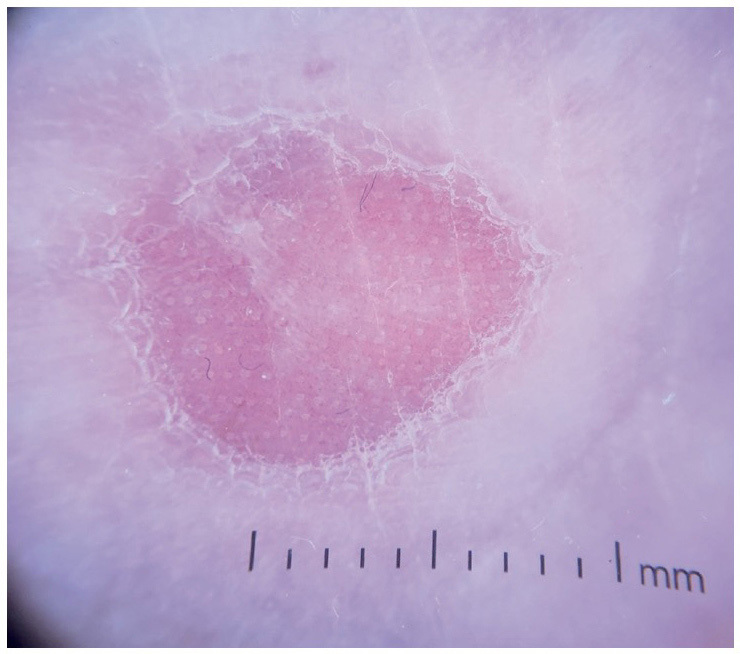
Dermoscopic image: Erythematous plaque with punctate vessels and regularly distributed white dots, delimited by a stepped-scaly border.

**Figure 3:**
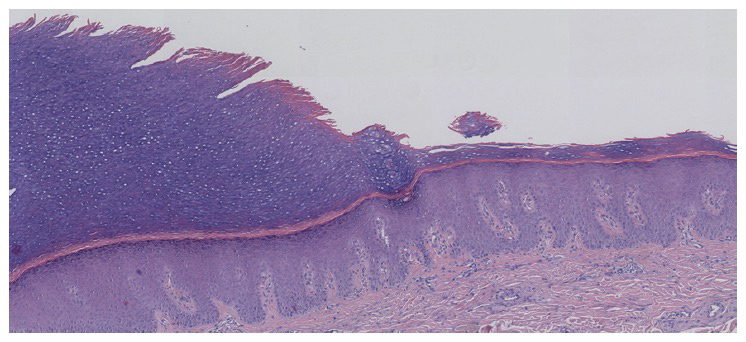
Skin structure formed by epidermis and dermis. Thinning of the epidermis with diminished stratum corneum and stratum granulosum and scattered mononuclear inflammatory elements in the dermis.

A 63-year-old woman, with a history of high blood pressure and Waldenström macroglobulinemia, presented to our department for the evaluation of a solitary well-demarcated depressed plaque in the thenar eminence of the left hand that had been present for 10 years (Figure 1). The patient denied any associated symptoms or previous trauma. Dermoscopy showed a stepped-scaly border and punctate vessels on an erythematous base, with regularly distributed white dots (Figure 2). A biopsy was performed that revealed a thinning of the stratum corneum and a slightly diminished stratum granulosum compared with the surrounding normal skin. Parakeratosis was not present, and only scattered mononuclear inflammatory elements were found in the dermis (Figure 3). The diagnosis of circumscribed palmar hypokeratosis was reached, and due to its benign nature, no treatment was carried out.

## Conclusions

Circumscribed palmar hypokeratosis is a little-known disease first described in 2002 by Urbina et al. It consists of an asymptomatic, erythematous, round-shaped, atrophic lesion, usually solitary, generally located in the thenar or hypothenar eminence of palms among middle-aged women [1]. The pathogenesis of this entity remains uncertain, but genetic abnormalities in keratin expression seem to be the main hypothesis, as a congenital case is described. Typical findings of dermoscopy correlate with histopathology, showing a peripheral stepped-scaly border delimiting an erythematous base due to the transparency of the stratum corneum with white spots probably representing acrosyringium. Histopathological findings are practically invariable, revealing an abrupt decrease of the stratum corneum compared with normal skin as the only particular characteristic in the histology. A subtly decreased granular layer is also frequent. Parakeratosis and cornoid lamella are usually absent. An unspecific inflammatory infiltrate in the dermis can be observed, though rarely [2]. Differential diagnosis of circumscribed palmar hypokeratosis should include porokeratosis of Mibelli, Bowen disease or traumatic erosions; however, the dermoscopy and histopathological findings are keys to reaching a correct diagnosis. We report a new case of this entity, that is infrequently seen but should be considered in the differential diagnoses of palm lesions.

## References

[1] Urbina F, Pérez A, Requena L, Rütten A (2014). Circumscribed palmar or plantar hypokeratosis 10 years after the first description: what is known and the issues under discussion. Actas Dermosifiliogr.

[2] Ishiko A, Dekio I, Fujimoto A, Kameyama K, Sakamoto M, Benno Y (2007). Abnormal keratin expression in circumscribed palmar hypokeratosis. J Am Acad Dermatol.

